# AIDS dementia complex and neuropsychiatric symptoms : a case report

**DOI:** 10.1192/j.eurpsy.2023.2039

**Published:** 2023-07-19

**Authors:** R. Fernández Fernández, P. del Sol Calderón, Á. Izquierdo de la Puente, A. Rodríguez Rodríguez

**Affiliations:** 1 Psychiatry, Hospital Universitario Infanta Cristina, Parla; 2 Psychiatry, Hospital Universitario Puerta de Hierro, Majadahonda; 3 Psychiatry, Hospital Universitario HM Puerta del Sur, Móstoles, Spain

## Abstract

**Introduction:**

HIV infection presents complications that may include neuropsychiatric symptoms and whose management is important to avoid misdiagnosis and mistreatment.

**Objectives:**

This case aims to highlight the importance of assessing HIV comorbidity in patients with psychiatric onset pathology.

**Methods:**

Case report and literature review.

**Results:**

We present the case of a patient diagnosed with HIV in 1985, who after 20 years of disease with irregular adherence begins to present delusional ideation of harm and self-referential, control experiences, thought diffusion phenomena, and possible auditory hallucinations, with poor evolution despite the establishment of numerous antipsychotic treatments, which evolves over the years towards a confabulatory character and with progressive neuropsychological deterioration. After numerous admissions, and despite several treatments, the patient developed over time memory failures, bradypsychia, gait disturbances, and difficulties in self-care, which further aggravated his condition by hindering therapeutic adherence, which ended with the patient’s chronic institutionalization. Diagnosis was AIDS dementia complex.

**Conclusions:**

HIV hardly replicates in the central nervous system but generates antigenemia which, in turn, generates an inflammatory infiltrate that can cause diffuse involvement, predominantly subcortical and limbic system. Usually, the dementia-AIDS picture is insidious and develops in patients with poor control of the primary disease. It is recommended to optimize antiretroviral therapy and neuroprotective agents, as well as symptomatic treatment by psychiatry.

**Disclosure of Interest:**

None Declared

